# Spin glass states in multicomponent layered perovskites

**DOI:** 10.1038/s41598-024-53896-2

**Published:** 2024-02-09

**Authors:** P. Pramanik, R. Clulow, D. C. Joshi, A. Stolpe, P. Berastegui, M. Sahlberg, R. Mathieu

**Affiliations:** 1 Department of Materials Science and Engineering, Uppsala University, Box 35, 751 03 Uppsala, Sweden; 2 Department of Chemistry - Ångström Laboratory, Uppsala University, Box 538, 751 21 Uppsala, Sweden; 3 FSCN Research Centre, Surface and Colloid Engineering, Mid Sweden University, 851 70 Sundsvall, Sweden

**Keywords:** Magnetic properties and materials, Phase transitions and critical phenomena

## Abstract

Temperature-dependent dc-magnetization and ac-susceptibility curves have been recorded for series of single and double layered *Ruddlesden-Popper* multicomponent perovskites with chemical formula A_2_BO_4_ and A_3_B_2_O_7_, respectively, with (La, Sr) on A-sites and up to 7 different cations on the B-sites (Ti, Cr, Mn, Fe, Co, Ni, Cu). The phase purity and chemical homogeneity of the compounds were investigated by X-ray diffraction and energy dispersive X-ray spectroscopy. Independently of the composition, spin glassiness is observed in both systems. Scaling analyses suggest the materials undergo spin glass phase transitions at low temperatures. Yet, qualitative differences are observed between the single-layered and double-layered systems, which are discussed in the light of the spatial dimensionality and magnetic interaction in layered oxide perovskites.

## Introduction

Multicomponent materials in the form of alloys and oxides have drawn a lot of attention over the years due to their distinct structural traits and correlated possibilities for customizing functional characteristics^1–9^. In a recent study, Rost et al. reported the possibility to combine five distinct cations in equi-atomic ratios to form a single-phase oxide system, which was defined as an “entropy-stabilized oxide” due to entropy-driven structural stabilization effect^3^. A more comprehensive term, “high-entropy oxides (HEOs)”, has been used to classify multi-cationic oxide systems; the systems' configurational entropy S_config_ (= R[y_i_lny_i_], where R and y_i_ stand for the Boltzman constant and percentage of each component, respectively), serves as the basis for the more all-encompassing concept of entropy stabilization^9–11^. As a result, by including many randomly distributed atoms on the same sites, the chemistry of entropy stability enables the creation of novel single-phased materials.

**Figure 1:**
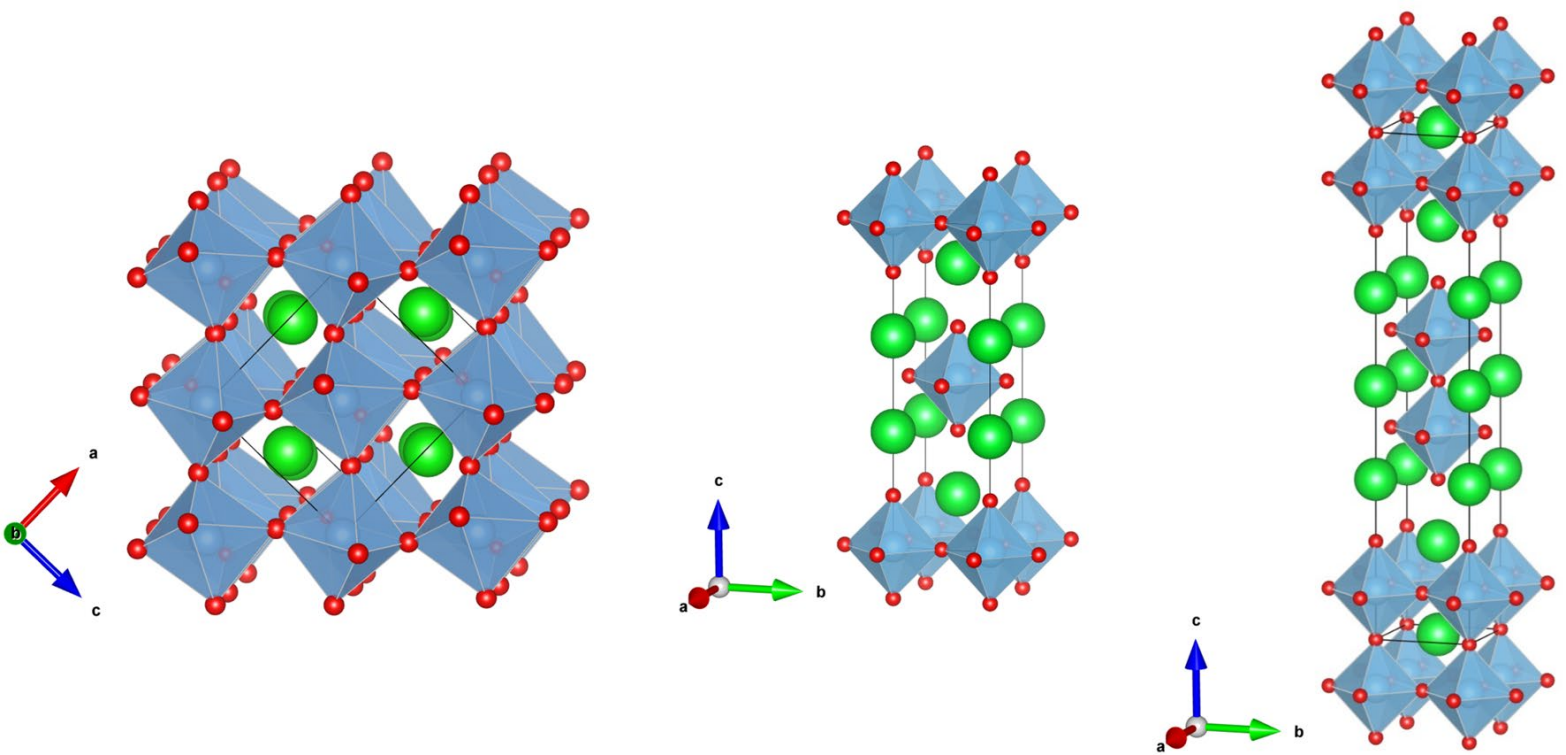
Polyhedral representations of the A_n + 1_B_n_O_3n + 1_ perovskites: (left) n = ∞; ABO_3_, labelled here "113"; (middle) n = 1; A_2_BO_4_, "214"; (right) n = 2; A_3_B_2_O_7_, "327″. Green spheres: non magnetic A-cations: blue: B-cations, red: oxygen anions; drawn using VESTA^36^.

Numerous HEOs including spinel, fluorite, perovskite, and rock salt crystal structures, have so far been created and investigated in order to modify their functional properties^12–15^. Jiang et al*.* were the first to describe a new family of multicomponent oxides with a perovskite structure^5^. Most of these materials show an antiferromagnetic/ferrimagnetic behavior^12,16,17^. Witte and co-authors also reported magnetic frustration in the rare earth (RE) and transition metal (TM) based high entropy oxides due to competing ferromagnetic and antiferromagnetic interactions in addition to the predominant antiferromagnetic coupling^12^. Spin glass states^18,19^ are known to be ubiquitous to magnetic perovskites, e.g. manganite perovskites^20^, more so in the low dimensional ones^20^. Because of the competing magnetic interactions between the sparsely dispersed cations, the magnetic order in HEOs is extremely complex. Spin glassiness could hence be expected from the complex mixture of cations in the lattice, yet no spin glass phase has been evidenced so far in those materials. Furthermore, little is known about the magnetic properties of lower dimensional multicomponent systems such as Ruddlesden-Popper perovskites with chemical formula A_n + 1_B_n_O_3n + 1_ (n = ∞: ABO_3_, labelled here "113"; n = 1: A_2_BO_4_, "214"; n = 2: A_3_B_2_O_7_, "327")^21^, which are illustrated in Fig. 1. Only a few reports are available, and mainly dealing with dimensionality-related superconducting properties^22^.

Here we report the magnetic properties of single and double layered Ruddlesden-Popper perovskites, which were found to show dynamical magnetic behavior and undergo spin glass phase transitions at low temperatures. We discuss the magnetic behavior of the glassy phases in the light of the magnetic interaction in these low-dimensional systems.

## Results and discussion

The multicomponent perovskites were synthesized via a solid-state reaction method, as described in detail in Refs.^17,23^. Two series of Ruddlesden-Popper perovskites consisting of single- and double-layer atomic arrangements, and chemical formulas A_2_BO_4_, and A_3_B_2_O_7_, respectively, were studied. B-sites are filled with up to seven different transition metal cations in order to increase the configurational tunability in term of ionic sizes and oxidation states^17^. La^3+^ and Sr^2+^ cations occupy the A-sites, and also affect the charge balance in the structure and the oxidation state of the B-site cations^17^. From here onwards the A_2_BO_4_ and A_3_B_2_O_7_ family of samples will be referred to as 214, and 327, respectively. In each series, a total of four samples with different compositions were prepared, denoted 214_N and 327_N, respectively (N = 1, 2, 3, 4); details of the chemical composition and labels are summarized in Table 1. For comparison a multicomponent perovskite with chemical formula La(Ti_1/7_Cr_1/7_Mn_1/7_Fe_1/7_Co_1/7_Ni_1/7_Cu_1/7_)O_3_ was prepared (labeled 113).

**Table 1:** Compositions of the different samples and their labels.

Phase	Chemical formula	Label
A(B)O_3_	La(Ti_1/7_Cr_1/7_Mn_1/7_Fe_1/7_Co_1/7_Ni_1/7_Cu_1/7_)O_3_	113
A_2_(B)O_4_	LaSr(Ti_1/7_Cr_1/7_Mn_1/7_Fe_1/7_Co_1/7_Ni_1/7_Cu_1/7_)O_4_	214_1
	LaSr(Ti_1/7_Mn_1/7_Fe_3/7_Co_1/7_Cu_1/7_)O_4_	214_2
	LaSr(Ti_1/7_Mn_2/7_Fe_1/7_Co_1/7_Ni_1/7_Cu_1/7_)O_4_	214_3
	LaSr(Cr_1/6_Mn_1/6_Fe_1/6_Co_1/6_Ni_1/6_Cu_1/6_)O_4_	214_4
A_3_(B)_2_O_7_	La_0.5_Sr_2.5_(Mn_1/4_Fe_1/4_Co_1/4_Ni_1/4_)_2_O_7_	327_1
	La_0.5_Sr_2.5_(Mn_1/5_Fe_2/5_Co_1/5_Ni_1/5_)_2_O_7_	327_2
	La_0.5_Sr_2.5_(Mn_2/6_Fe_2/6_Co_1/6_Ni_1/6_)_2_O_7_	327_3
	La_0.5_Sr_2.5_(Ti_1/5_Mn_1/5_Fe_1/5_Co_1/5_Ni_1/5_)_2_O_7_	327_4

The crystal structure of the samples from each family were obtained from powder X-ray diffraction using the Rietveld refinement method (see Methods and supplementary Figs. SM1–SM3). The 113 sample adopts an orthorhombic structure with space group *Pnma* while the 214 and 327 perovskites adopt a tetragonal crystal structure with *I4/mmm* space group. The lattice parameters obtained from Rietveld refinements are listed in Table SM1. The lattice parameters obtained for the 113 sample are comparable to those reported earlier^17^. It is interesting to note that the *c/a* ratios between *c*- and *a*-axis lattice parameters are greater than 3 and 5 for the 214 and 327 series, respectively (see also Fig. 1). The surface morphology and individual elemental mapping for different atoms present in each phase were determined by the EDS analysis and are shown in Figs. SM7-SM8, respectively. The results of the EDS chemical compositional analysis obtained from ten various surface sites indicate average cationic compositions close to the expected nominal compositions, e.g. LaSr_1.04_(Ti_0.13_Cr_0.13_Mn_0.15_Fe_0.14_Co_0.17_Ni_0.14_Cu_0.10_)O_4_ and La_0.53_Sr_2.47_(Mn_0.52_Fe_0.50_Co_0.51_Ni_0.48_)O_7_, for LaSr(Ti_0.14_Cr_0.14_Mn_0.14_Fe_0.14_Co_0.14_Ni_0.14_Cu_0.14_)O_4_ and La_0.5_Sr_2.5_(Mn_0.5_Fe_0.5_Co_0.5_Ni_0.5_)O_7_, respectively. EDS maps and results are given in the Supplemental Materials for a 214 phase (Figs. SM7 and SM8 and Table SM2); see Ref. ^9^ for the 113 phase and Ref.^23^ for the 327 ones.

**Figure 2:**
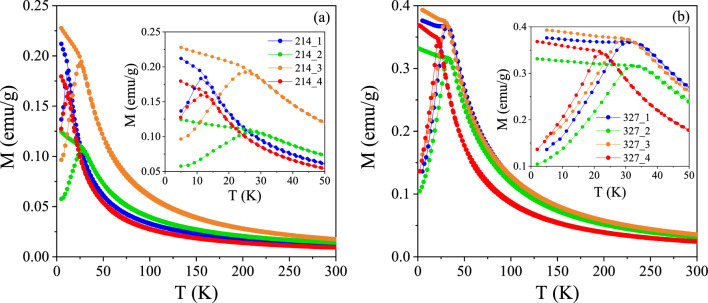
ZFC/FC magnetization M recorded in H = 1000 Oe for the (**a**) 214 and (**b**) 327 systems. The insets show the low-temperature behavior in more detail.

**Figure 3:**
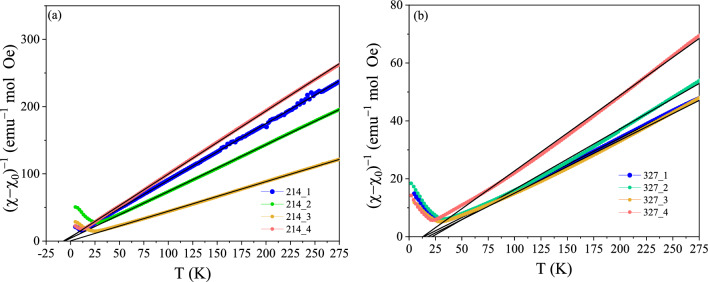
Temperature dependence of the inverse susceptibility 1/χ =  H/M vs T (H = 1000 Oe; filled markers) and linear fits (lines) for (**a**) 214 and (**b**) 327 systems, implying χ =  C/(T−θ_CW_) + χ_o_ (see Table SM3).

The temperature-dependent zero-field-cooled (ZFC) and field-cooled (FC) magnetization M(T) of the investigated 214 and 327 systems recorded in H = 1000 Oe is presented in Fig. 2. They all exhibit a cusp in the ZFC magnetization below 40 K, while the FC magnetization is found to slightly increase with decreasing temperature; more significantly in the 214 case. No magnetic irreversibility is observed above the cusp temperature. While the temperature onset of the cusp is different by several degrees, the ZFC/FC curves of all the 214 (resp. all the 327) samples are qualitatively similar, and thus rather independent of the composition. The FC magnetization data is plotted as the inverse susceptibility H/M = 1/χ vs T in Fig. 3 to investigate the Curie–Weiss behavior. A linear behavior was obtained in all cases down to the lowest temperatures above the cusp, suggesting a homogeneous magnetic response. Lower (in absolute values) Curie–Weiss temperatures θ_CW_ were obtained for the 214 phases (~ 0–7 K vs ~ 13–22 K for the 327 ones; see Table SM5). Note that here we have aimed at showing the qualitatively similar magnetic behavior of the samples (see M(T) or χ(T) curves), relatively independently of their composition, rather than correlating the values of the Curie–Weiss temperatures and associated effective moments to compositions; those parameters are listed in Table SM3. Magnetic field dependent M(H) data was also collected and is shown for reference in the Supplemental Materials (Fig. SM10). The magnetic behavior evidenced by the ZFC/FC curves in Fig. 2 is reminiscent to that of spin glasses^18,19,24^. Interestingly such spin glass states are ubiquituous in layered perovskites such as layered manganites^20,25^. However, in order to evidence magnetic glassiness, and a low-temperature spin glass state, it is important to evidence that the material displays typical glassy characteristics such as aging, memory, and rejuvenation^18,24^ at low temperatures, and undergoes a spin glass phase transition^18,20^.

If a spin glass is quenched from a temperature above its spin glass phase transition temperature T_g_ to a halt temperature below T_g_ in the glassy phase, and kept at that temperature for a certain time, the spin configuration established during cooling will slowly equilibrate by rearranging itself, or age^24^. Interestingly, this aging may at the same time be "kept in memory" by the system (memory feature) and forgotten (rejuvenation) on further temperature changes, as the spin configuration is rearranged on shorter length scales^24^. This implies that if the system is further cooled down to a temperature far enough, it will appear rejuvenated (i.e. as if no aging had occurred), yet its aged configuration will be recovered upon reheating to the halt temperature^24^. As a result, a relatively simple way to probe the glassy behavior, assuming magnetic fields low enough to provide a linear response, is to perform so-called memory experiments^24^. In such experiments, the ZFC magnetization is first recorded on reheating in a small magnetic field after cooling the material from a reference temperature above T_g_ down to the lowest temperature (reference curve). The ZFC magnetization is then acquired one more time, now including a halt (or several) at a constant temperature T_h_ in the glassy phase for a certain halt time t_h_, during the cooling to the lowest temperature (memory curve). In both cases, the magnetic field is always zero during the cooling and waiting. The aging at the halt temperature and subsequent rejuvenation on further cooling will have the memory curve coinciding with the reference curve at all temperatures but in the vicinity of T_h_ ; defining a "memory dip" in the (memory minus reference) difference curves^17^.

**Figure 4:**
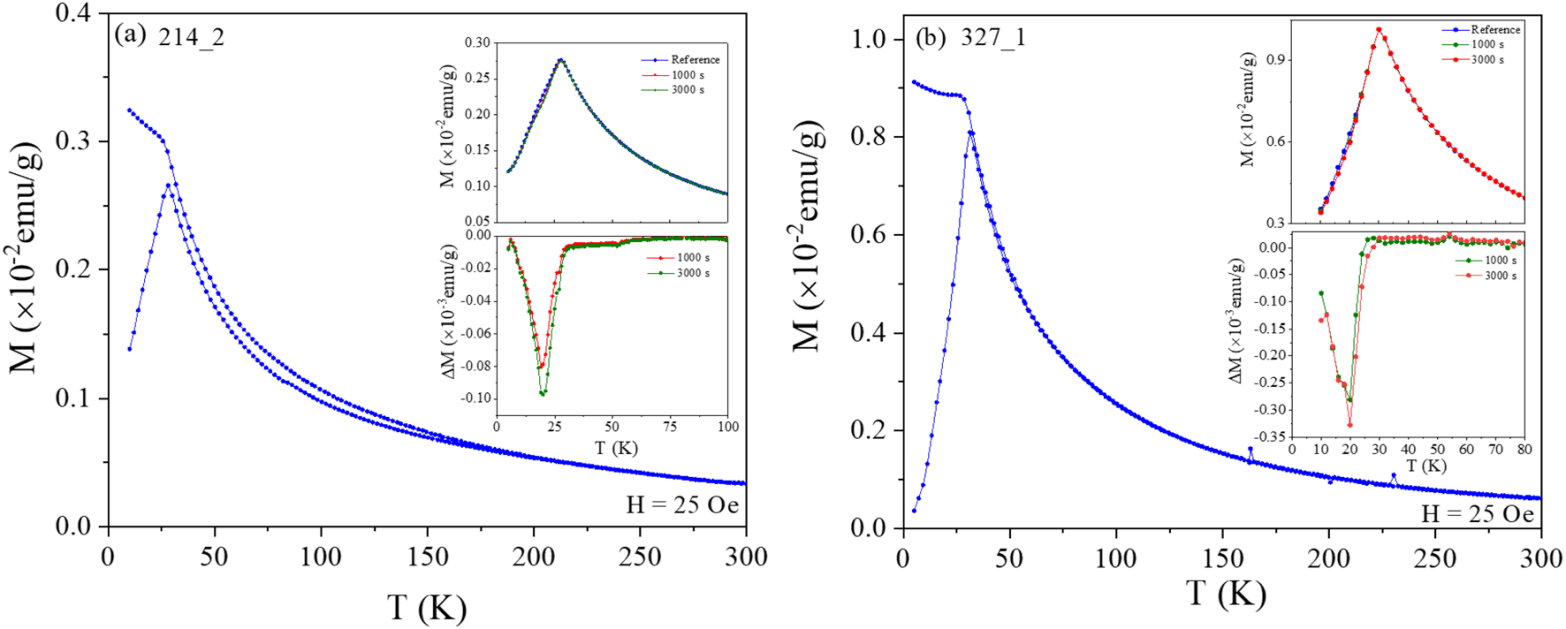
ZFC/FC magnetization recorded in H = 25 Oe for the (**a**) 214 and (**b**) 327 systems. The insets show the results of memory experiments: (top) ZFC magnetization recorded in H = 25 Oe with and without halts at T = 20 K in the cooling and (bottom) their corresponding difference curves ΔM versus T (curve with halt minus reference curve without halt).

The ZFC/FC curves of two 214 and 327 systems recorded in a small field (H = 25 Oe) are shown in Fig. 4. The 327 phase display no irreversibility above the cusp temperature, while a slight irreversibility is observed for the 214 system, which may reflect a minor contribution from a secondary 113 phase (see Fig. SM2). Memory experiments were performed on both systems, including halts of t_h_ = 1000 s and 3000 s at T_h_ = 20 K. As seen in the insets of the Figure, both systems exhibit the characteristic memory dips associated with a glassy phase, and no significant features above the ZFC cusp temperature. In order to check whether the systems undergo a spin glass phase transition, ac-susceptibility measurements were performed and analyzed. First, it is interesting to note that spin glass-like ZFC/FC curves akin to those displayed by the 214 and 327 systems investigated here have not been observed in three dimensional (113) multicomponent perovskites. In the 113 systems which do not show a long ranged ferrimagnetic/antiferromagnetic order, ZFC/FC curves akin to those presented in Fig. SM9(a) have been reported^17,26^, with a broad cusp in the ZFC magnetization, and magnetic irreversibility above the cusp temperature, even in relatively large magnetic fields (here 1000 Oe). This kind of magnetic behavior is often attributed to spin glass states, without further study^18^, and may instead reflect the magnetic inhomogeneity of the system, e.g. the inhomogeneous distribution of the magnetic cations on the B-site. As seen in Fig. SM9(b), memory dips are observed in the ZFC memory curves of the 113 sample recorded in a small magnetic field, indicating a glassy behavior. However the lack of well defined onset of non-equilibrium dynamics (e.g. a cusp, akin to that obtained in larger fields) suggests a lack of spin-glass phase transition, and hence that the observed dynamical magnetic behavior reflects the magnetic inhomogeneity of the system^20,27^. Note that the 75–200 K region does not seem to be solely paramagnetic, considering the magnetization data in high and low magnetic fields presented in Fig. SM9 (a) and (b).

**Figure 5:**
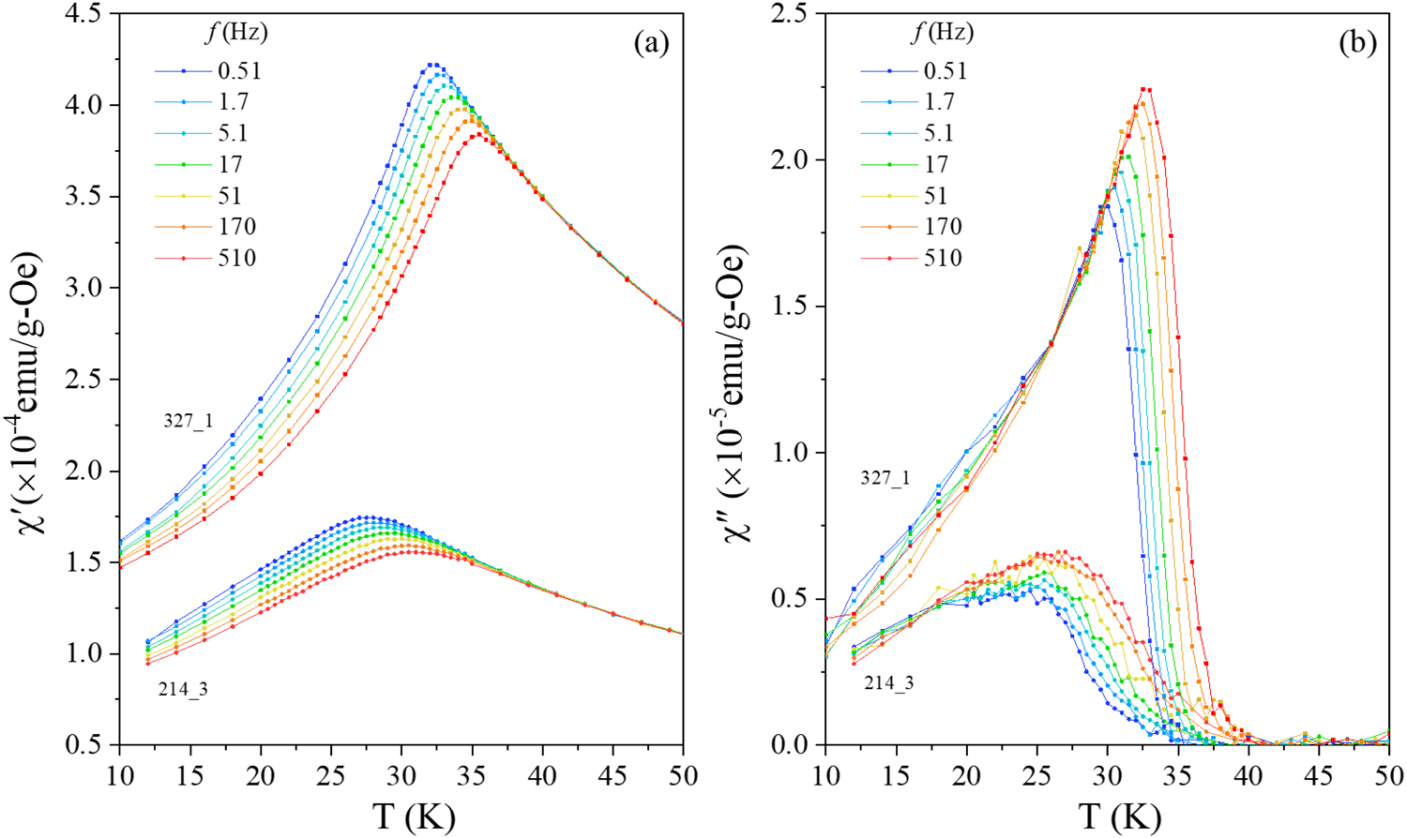
Temperature dependence of the (**a**) in-phase χ′(ω = 2πf,T) and (**b**) out-of-phase χ′′ (ω,T) components of the ac-susceptibility for selected 214 and 327 samples recorded using a small ac-excitation h = 4 Oe for different frequencies f.

**Figure 6:**
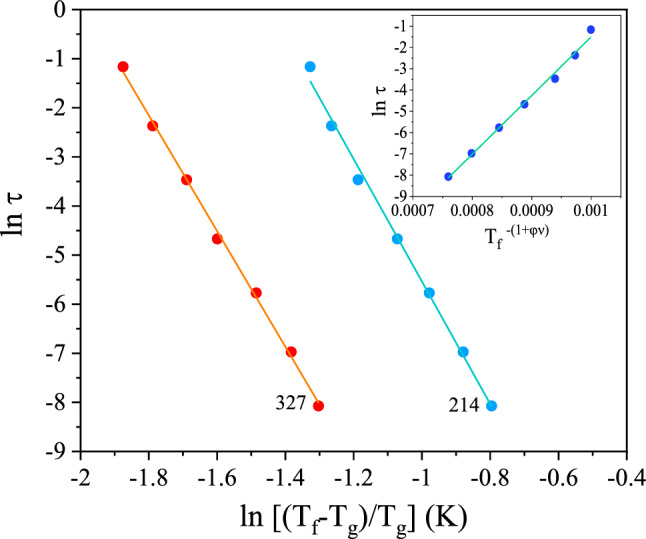
(3D) Scaling of τ with the reduced temperature ϵ = (T−T_g_)/T_g_ for both 327 and 214 systems. A 2D scaling (T_g_ = 0) is attempted in the inset for the 214 sample.

The ac susceptibility curves of selected 214 and 327 systems are shown in Fig. 5. The temperature and frequency dependence of those curves resemble those of spin glasses in both cases^18,28^. χ'(ω,T) curves "depart" from the equilibrium curve at lower and lower temperatures as the frequency of the ac excitation (h = 4 Oe, f = ω/2π = 0.51 Hz to 510 Hz) decreases. This corresponds for χ"(ω,T) to a frequency dependent onset which shifts accordingly in temperature^18,28^. The onset of non-equilibrium is sharp in the 327 case, which suggests a spin glass phase transition. The cusp in the χ'(ω,T) curves appear broader (and noisier due to the low magnetic signal) in the 214 case and the temperature onset of χ"(ω,T) is not as sharp. A dynamical scaling analysis is performed in order to check the critical slowing down at the phase transition^18,19^. Each frequency in the ac measurement defines a timescale, or observation time of the measurement, τ ~ 1/ω. Freezing temperatures T_f_ marking the onset of non equilibrium dynamics can be estimated from the susceptibility curves for each frequency, yielding (τ,T_f_) datasets which, in case of a spin glass phase transition exhibit the power law τ = τ_0_ε^-zν^ where ε = (T_f_−T_g_)/T_g_ is the reduced temperature, z and ν critical exponents, and τ_0_ reflects the microscopic flipping time of the interacting entities^18,28^. As seen in the main frame of Fig. 6, a relatively good scaling of the τ(ε) is observed for both the 214 and 327 phases, yielding physical values of the zν product and τ_0_ : zν ~ 12 ± 1 both for 214 and 327 phases and τ_0_ ~ 10^–8±1^ s for the 214 and ~ 6 × 10^–11±1^ s for the 327 phase, respectively (T_g_ ~ 25 ± 1 K and 29 ± 1 K respectively), suggesting that the systems undergo a spin glass phase transition. While the obtained values of the zν product are compatible with those of anisotropic spin glasses^18,28^ in the 10–12 range for both systems, the values of τ_0_ are larger than that observed for model (atomic) spin glasses in the 214 case. Larger τ_0_ values are typical for so-called superspin glasses, i.e. spin glasses in which the interacting entities are not single, atomic, spins, but superspins comprising several (many) coherently arranged spins; the amount determining the time scale of the fluctuation, i.e. τ_0_^29^. Such superspin glasses are often observed in strongly interacting magnetic nanoparticle systems^29^, and have also been evinced in bulk systems whose magnetic state is build up of small (ferromagnetic) entities such as certain transition metal oxide perovskites^20^; there τ_0_ ~ 10^–11±1^ s (e.g. 113:^30^, 214:^20,31^).

In the 214 perovskites, spin glass states with lower (2D) spatial dimensionality have been observed^32^. Considering the 2D generalized Arrhenius scaling form for the slowing down, log(τ/τ_0_) = T_f_^-(1+ψν)^ where ψ is another exponent^20,32^, yields acceptable fits for the 214 phase, setting ψν to its "maximal" value of 1^32^; implying τ_o_ ~ 6 × 10^–13±1^ s (see inset of Fig. 6). In the 2D case, the scaling form of the χ"(ω,T) is relatively simple^20,32^ and the full scaling of the χ"(ω,T) data may be attempted^32^. As seen in Fig. SM12, a good scaling is observed for the 214 data using the values of ψν and τ_0_ obtained from the τ(T_f_) scaling.

## Conclusions

The dynamical magnetic properties of two series of novel low-dimensional *Ruddlesden-Popper* multicomponent perovskites were investigated in detail. Zero-field cooled memory experiments evidence magnetic glassiness in both single layered (“214”) and double layered (“327”) systems at low temperatures. Scaling analyses of the ac-susceptibility suggest that both systems undergo a (3D) spin glass phase transition at a finite T_g_, albeit qualitative differences can be observed between the 214 and 327 phases, possibly related to spatial dimensionality and magnetic interaction. In the 214 case, a reasonable scaling of the ac-susceptibility data is obtained considering a (2D) spin glass phase transition at T_g_ = 0 K.

## Methods

The phase purity and crystal structure of all the sintered samples were determined by performing X-ray powder diffraction on a Bruker D8 ADVANCE diffractometer equipped with a Lynx-eye XE position sensitive detector (PSD) using CuK_α_ radiation (λ = 1.5418 Å). The diffraction patterns were collected at room temperature in the 10—100° range in step of 0.014°. The data were analyzed using the Rietveld refinement method^33^ within the Topas 6 program^34^. Synchrotron X-ray diffraction data of the 214 samples were measured at the P02.1 beamline at PETRA III (λ = 0.207 Å). The data were recorded using a PerkinElmer XRD1621 fast area detector and integrated to a 1D diffraction pattern using the software Fit 2D^35^. Polyhedral representations were drawn using VESTA^36^. By collecting energy-dispersive X-ray spectroscopy (EDS) data from various locations on the sample surface, the elemental compositions of single-phase compounds were examined. Using a superconducting quantum interference device (SQUID) magnetometer from Quantum Design Inc., the dc-magnetization (M) and ac-susceptibility χ(ω = 2πf,T) data was collected and used to analyze the static and dynamical magnetic properties of sintered pellets. In the ac experiments, frequencies (*f)* ranging from 0.51 to 510 Hz and ac excitation h = 4 Oe were employed. Furthermore, the aging and rejuvenation-like properties of selected samples were investigated by performing memory experiments following the dc-memory protocol^24^. Magnetic field (H) dependent magnetization measurements for all samples were recorded at the lowest temperature.

### Supplementary Information

Supplementary Information.

## Data Availability

The datasets used and/or analysed during the current study available from the corresponding author on reasonable request.
