# Supplementary material for: Spin glass states in multicomponent layered perovskites
Source: Sci Rep. 2024 Feb 9;14:3382. doi: 10.1038/s41598-024-53896-2 (PMC10858224; doi:10.1038/s41598-024-53896-2)
Supplement: Supplementary file 1 — Supplementary Information. [file 41598_2024_53896_MOESM1_ESM.pdf]

Supplemental Materials

For

Spin Glass States in multicomponent layered perovskites

P. Pramanik[1,*], R. Clulow[2], D. C. Joshi[1], A. Stolpe[2,3], P. Berastegui[2], M. Sahlberg[2], R. Mathieu[1,*]

1 Department of Materials Science and Engineering, Uppsala University, Box 35, 751 03 Uppsala, Sweden

2 Department of Chemistry - Ångström Laboratory, Uppsala University, Box 538, 751 21 Uppsala, Sweden

3 FSCN Research Centre, Surface and Colloid Engineering, Mid Sweden University, 851 70 Sundsvall, Sweden

The Supplemental Materials include:

1. X-ray powder diffraction (XRPD) results: Diffractograms, Rietveld refinements and extracted lattice parameters for the 113 phase and selected 214 and 327 systems.

2. Energy-dispersive X-ray spectroscopy (EDS) results for a 214 system: image, maps, extracted cation contents.

3. Magnetic results: ZFC/FC curves for the 113 system; Curie-Weiss parameters for all the 214 and 327 systems; M(H) data for all the 214 and 327 systems; “full” 2D scaling for 214_3.

1. XRPD results

1.1 XRPD data and Rietveld refinements for selected samples

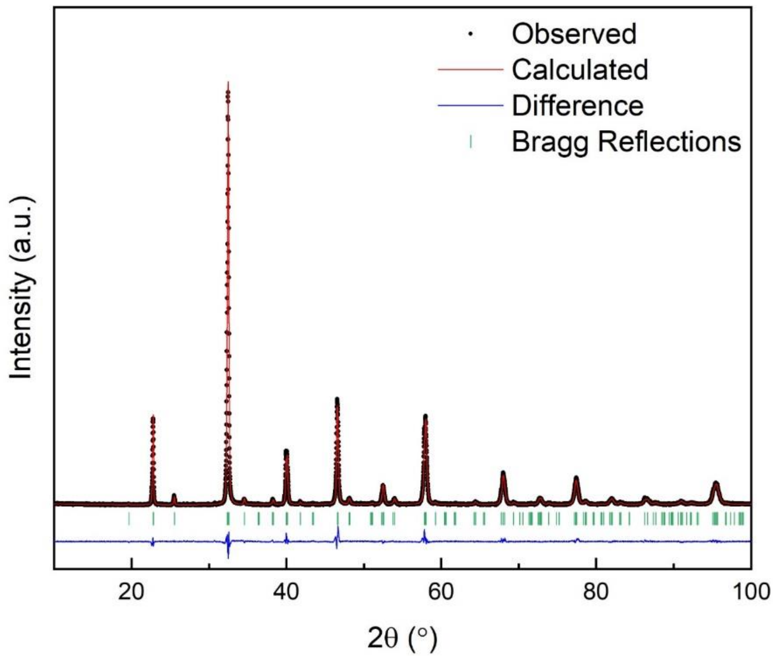

Fig. SM1 XRPD data and refinements for the 113 sample; R_{wp} = 5.80, χ^2 = 3.19.

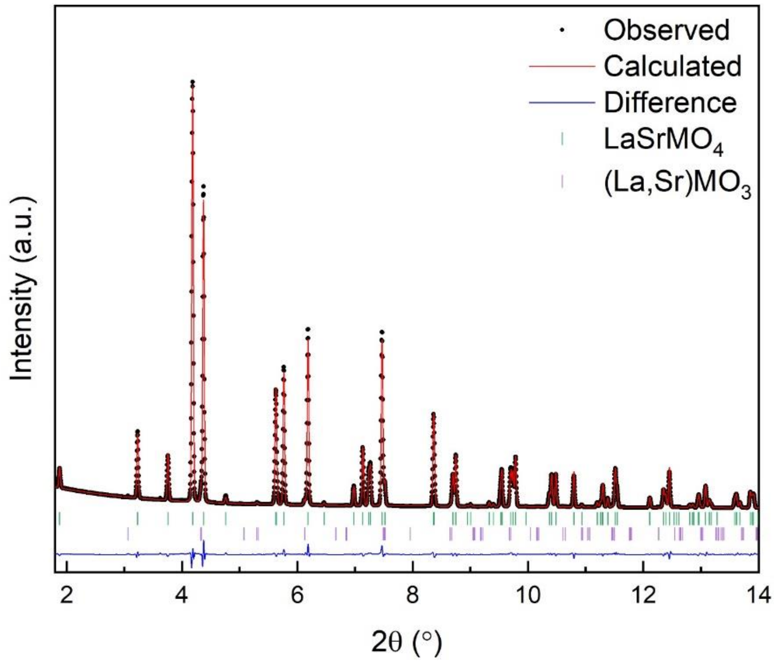

Fig. SM2 (Synchrotron) XRPD data and refinements for the 214_1 sample; R_{wp} = 2.83, χ^2 = 2.97. A $La_{1-x}Sr_xMO_3$ secondary phase, denoted **(**La,Sr)MO_3 in the figure, with an unknown x, was considered in the refinements (~ 2 wt%, space group R$\bar{3}$c, *a* = 5.4882(5) Å, *c* = 13.3853(30) Å). This secondary phase was also detected in similar amount in 214_2, but not for the 214_3 and 214_4 samples.

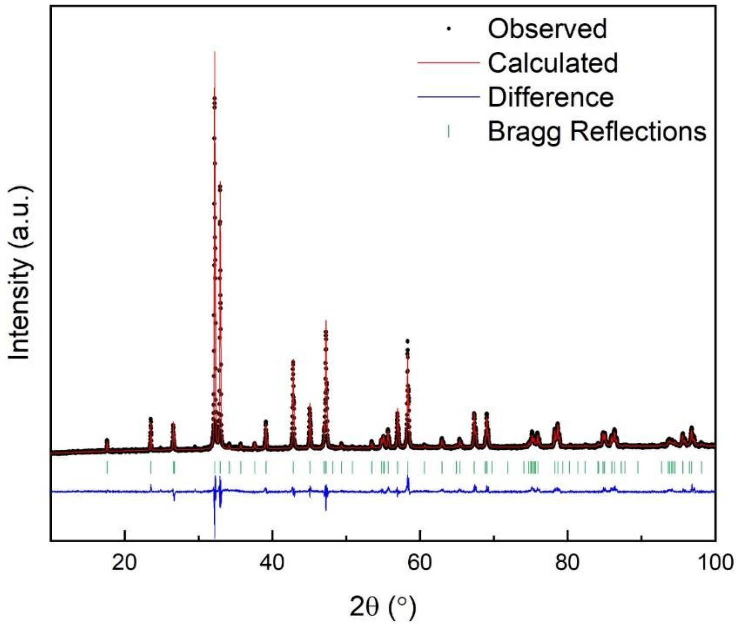

Fig. SM3 XRPD data and refinements for the 327_1 sample; R_{wp} = 5.81, χ^2 = 4.45.

1.2 Lattice parameters for all samples

Table SM1. Space groups and lattice parameters extracted from the Rietveld refinements of the XRPD data presented in Figs. SM1-SM3 and Ref. 23. * denotes synchrotron XRPD data. A Pawley refinement of the 214_4 data (denoted 214_4 (P)) was also performed as no high-quality synchrotron data is available for that sample. Refinement quality factors are also included.

		Lattice parameters (Å)				
Sample	Space group	*a*	*b*	*c*	R_{wp}	χ^2
113	*Pnma*	5.4985(1)	7.7846(1)	5.5287(1)	5.80	3.19
214_1 *	*I4/mmm*	3.8372(1)		12.6302(1)	2.83	2.97
214_2 *		3.8480(1)		12.7202(1)	4.18	6.2
214_3 *		3.8401(1)		12.6767(1)	3.78	4.94
214_4		3.8308(1)		12.6649(1)	18.1	11.29
214_4 (P)		3.8308(1)		12.6649(1)	10.511	3.83
327_1	*I4/mmm*	3.8438(1)		20.0972(3)	5.81	4.45
327_2		3.8490(1)		20.1211(4)	6.44	4.39
327_3		3.8465(1)		20.1215(3)	7.28	4.41
327_4		3.8551(1)		20.1646(4)	7.51	5.57

1.3 Additional XRPD data for the 214 samples

For XRPD results on all the 327 samples, see Ref. 23 (*R. Clulow et al., Phase stability and magnetic properties of compositionally complex n=2 Ruddlesden Popper perovskites, Submitted).*

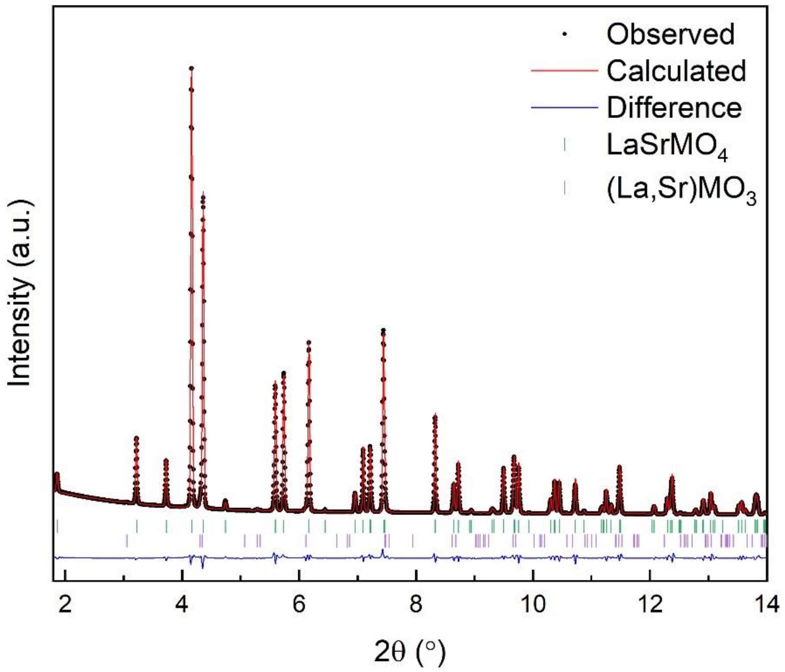

Fig. SM4 XRPD data and refinements for the 214_2 sample; R_{wp} = 4.18, χ^2 = 6.2.

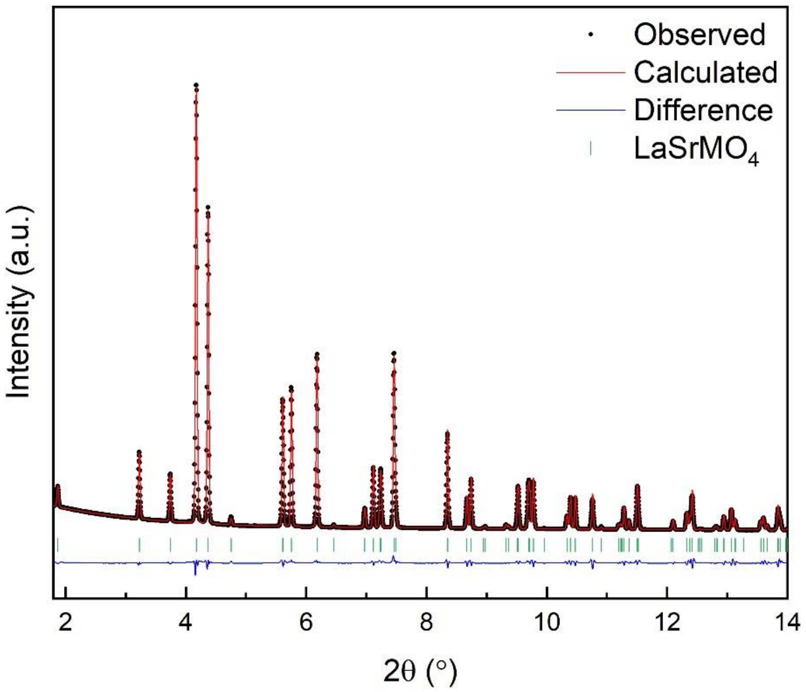

Fig. SM5 XRPD data and refinements for the 214_3 sample; R_{wp} = 3.78, χ^2 = 4.94.

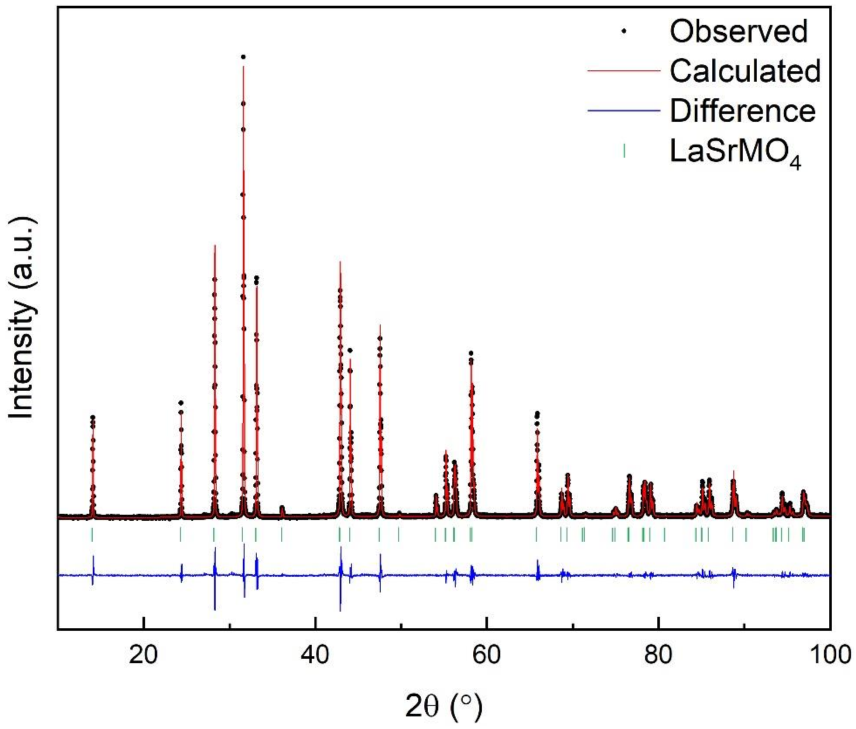

Fig. SM6 XRPD data and Pawley refinements for the 214_4 sample; R_{wp} = 10.511, χ^2 = 3.83.

2. EDS results

2.1 EDS images and maps for 214_1

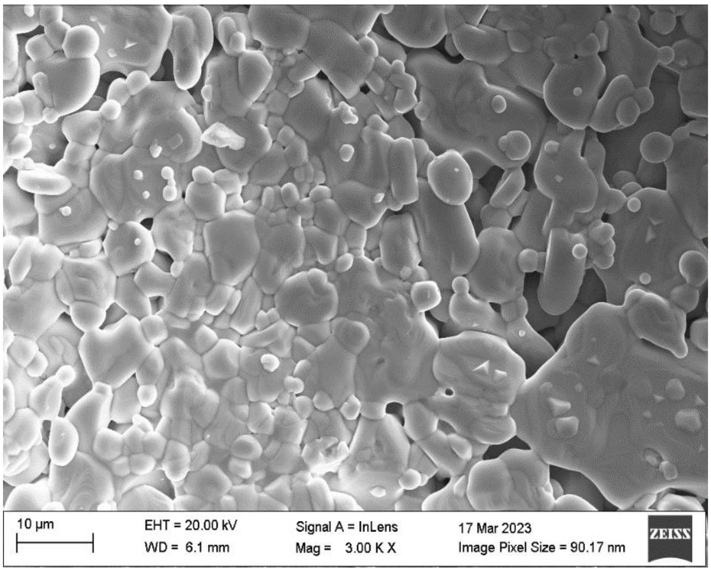

Fig. SM7. EDS image for 214_1

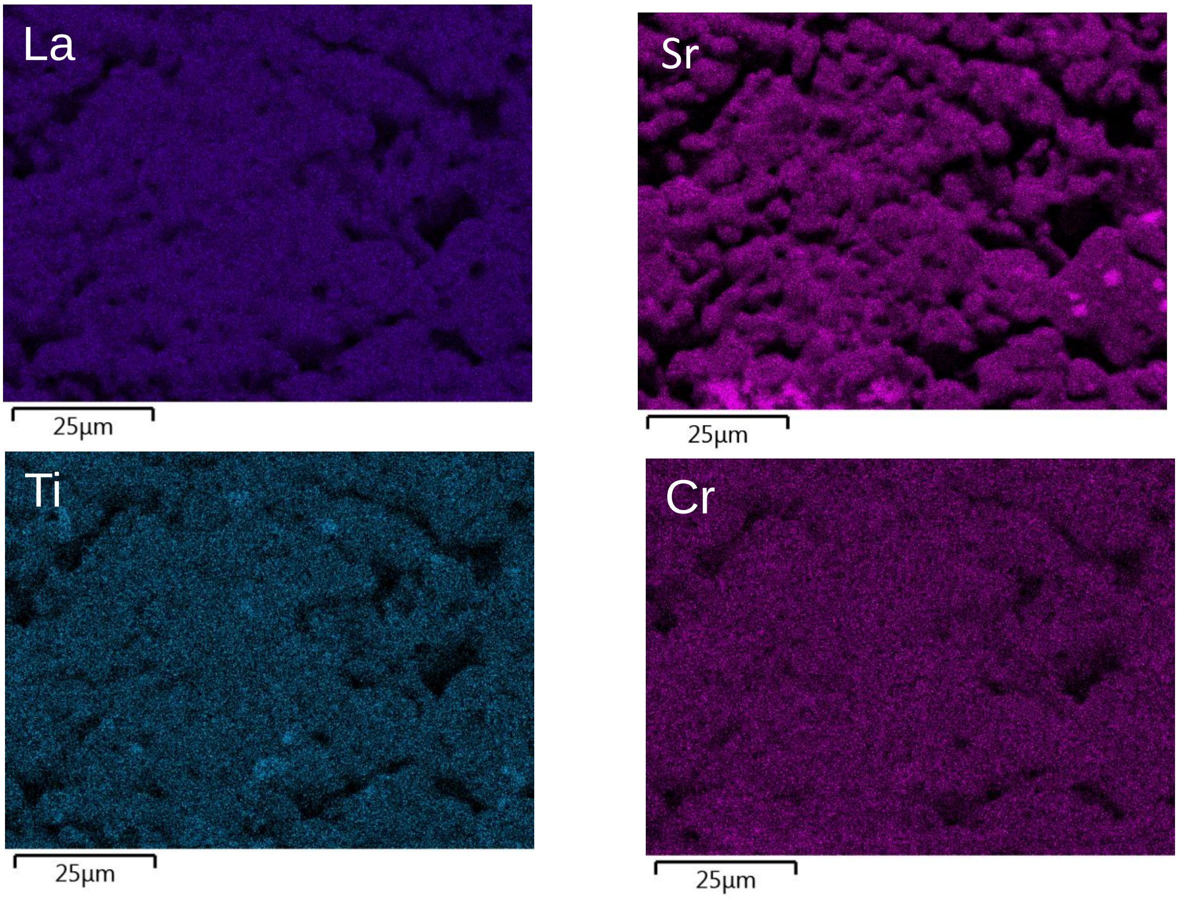

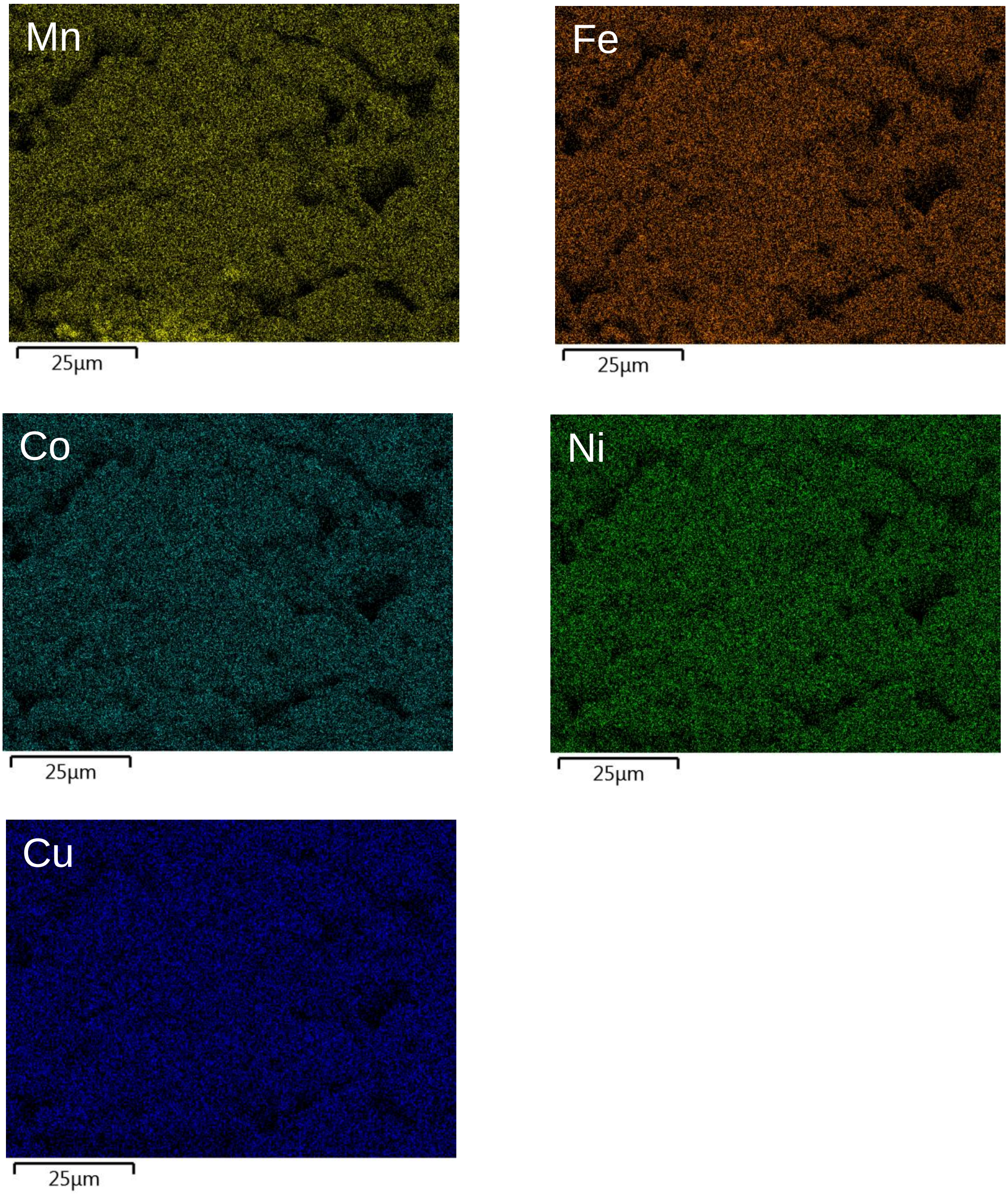

Fig. SM8. EDS maps for 214_1

2.2 Cation contents for 214_1

214_1	Ti	Cr	Mn	Fe	Co	Ni	Cu	Sr	La	Total
Spectrum 1	4.96	4.32	4.06	4.62	5.48	4.24	3.39	36.74	32.19	100
Spectrum 2	4.37	4.02	4.4	4.88	5.34	4.57	3.06	37.53	31.83	100
Spectrum 3	4.17	4.33	4.92	4.36	5.41	4.45	3.24	37.07	32.06	100
Spectrum 4	4.45	4.22	4.58	4.19	5.82	4.75	3.64	36.35	31.99	100
Spectrum 5	4.34	3.99	5.7	4.59	6.06	4.93	3.29	32.49	34.61	100
Spectrum 6	4.06	4.22	5.68	4.01	5.56	4.29	3.32	37.3	31.56	100
Spectrum 7	4.35	4.38	4.31	4.67	5.85	4.67	4.36	34	33.41	100
Spectrum 8	4.3	4.95	5.96	5.36	6.29	4.86	3.83	27.47	36.98	100
Spectrum 9	4.14	4.97	4.69	4.93	5.29	4.04	3.09	35.72	33.12	100
Spectrum 10	4.68	4.8	4.72	5.03	6.3	4.81	3.29	31.76	34.61	100
Max	4.96	4.97	5.96	5.36	6.3	4.93	4.36	37.53	36.98	
Min	4.06	3.99	4.06	4.01	5.29	4.04	3.06	27.47	31.56	
**Average**	**4.38**	**4.42**	**4.9**	**4.66**	**5.74**	**4.56**	**3.45**	**34.64**	**33.24**	
**Standard Deviation**	**0.27**	**0.36**	**0.66**	**0.41**	**0.38**	**0.3**	**0.39**	**3.24**	**1.72**	

Table SM2. Extracted cation contents, yielding the stoichiometry: $La_{1.00}Sr_{1.04}(Ti_{0.13}Cr_{0.13}Mn_{0.15}Fe_{0.14}Co_{0.17}Ni_{0.14}Cu_{0.10})O_4$ (nominal: $LaSr(Ti_{1/7}Cr_{1/7}Mn_{1/7}Fe_{1/7}Co_{1/7}Ni_{1/7}Cu_{1/7})O_4$).

3. Magnetic data

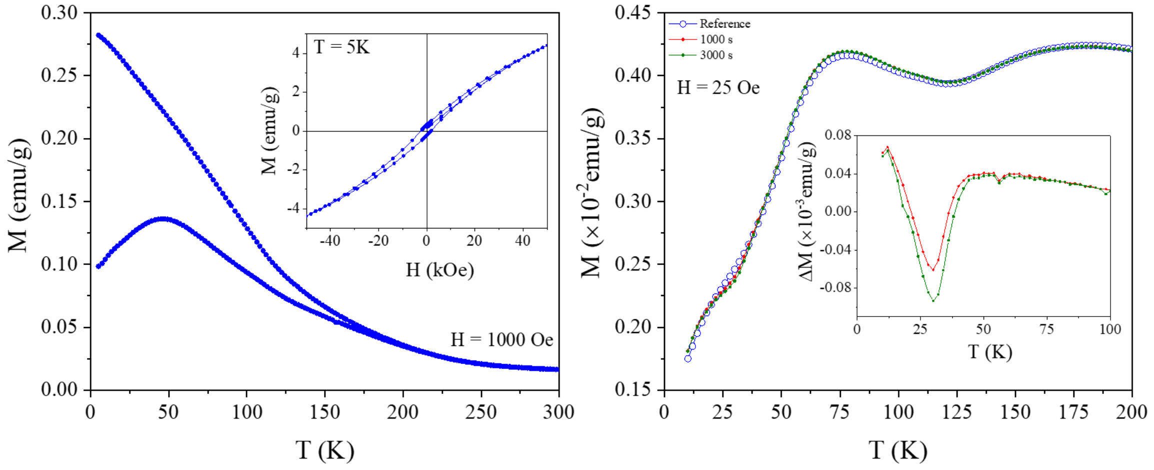

Fig. SM9. (a) ZFC/FC magnetization recorded in H = 1000 Oe for the 113 material. Inset shows M vs. H at 5K. (b) ZFC magnetization recorded in H = 25 Oe with and without halt at T = 30 K in the cooling. The inset shows the corresponding difference curves ΔM vs. T (curve with halt minus reference curve without halt).

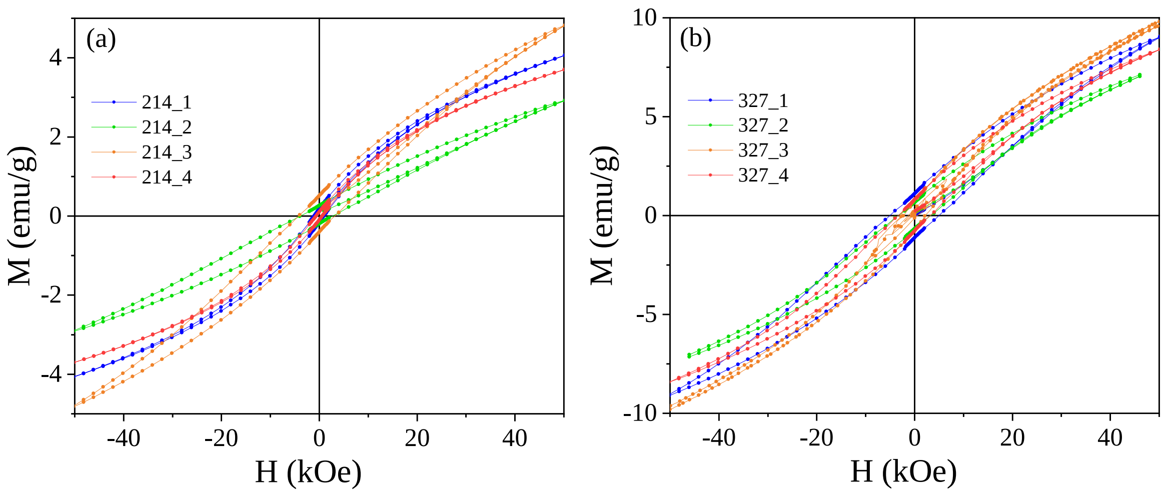

Fig. SM10: Magnetization vs magnetic field hysteresis curves at T = 5 K for (a) 214 and (b) 327 systems.

Table SM3. List of Curie-Weiss fitting parameters (θ_{CW}, μ_{eff}, and χ_o) for the 214 and 327 systems.

Sample	θ_{CW} (K)	μ_{eff} (μ_B/f.u.)	χ_o (emu/mol.Oe)
214_1	-6.9	3.1	5×10^{-4}
214_2	-5.2	3.4	2×10^{-4}
214_3	0	4.25	-1.5×10^{-3}
214_4	-5.8	2.97	-3×10^{-4}
327_1	14.5	6.6	-4×10^{-3}
327_2	22	6.18	-5.5×10^{-4}
327_3	18.4	6.6	-6×10^{-4}
327_4	13.3	5.53	-6×10^{-4}

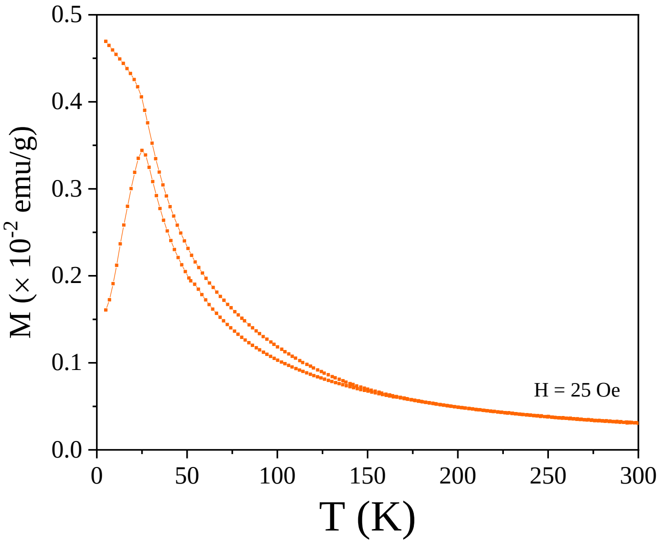

Fig. SM11: ZFC/FC magnetization curves recorded in H = 25 Oe for 214_3 (used in ac studies).

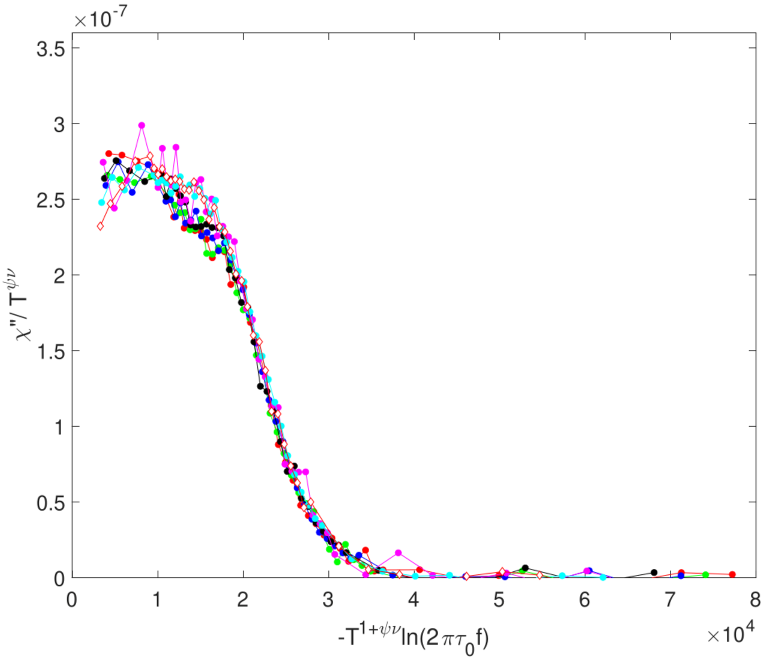

Fig. SM12. 2D dynamical scaling of the χ''(ω,T) data collected for the 214_3 sample.
